# ﻿*Desmopuntius mahakamensis*, a new cyprinid species (Teleostei, Cyprinidae) from East Kalimantan, Indonesia

**DOI:** 10.3897/zookeys.1256.158411

**Published:** 2025-10-23

**Authors:** Tonisman Harefa, Haryono Haryono, Rudhy Gustiano, Tedjo Sukmono, Gema Wahyudewantoro

**Affiliations:** 1 Research Center for Biosystematics and Evolution, National Research and Innovation Agency, Cibinong, 16911, Indonesia Research Center for Biosystematics and Evolution, National Research and Innovation Agency Cibinong Indonesia; 2 Department of Biology, Universitas Jambi, Jambi, Sumatra, 36122, Indonesia Universitas Jambi Jambi Indonesia

**Keywords:** Biodiversity, Borneo, *COI*, DNA barcoding, freshwater fish, integrative taxonomy, stripe-bodied barb, systematics

## Abstract

*Desmopuntius mahakamensis***sp. nov.**, is described based on specimens from East Kalimantan, Indonesia. The new species, *D. mahakamensis* is distinguished from all congeners by the following combination of characters: anal-fin rays iii,5½; lateral line scales 26–27 (mode 26); predorsal scales 9–11 (mode 10); gill rakers 9–11 (mode 10); total vertebrae 29; axial streak present; presence of 5–6 black lateral stripes in adults (>30 mm SL), with stripe +1 extending from behind the gill opening along scale row 1 and reaching only to midbody, between the origin and end of the dorsal-fin base. Molecular evidence further supports the assignment of the new species in the genus *Desmopuntius* and its clear separation from congeners.

## ﻿Introduction

The freshwater fish genus *Desmopuntius* Kottelat, 2013, comprises relatively small to medium-sized cyprinids that are widely distributed in Southeast Asia, including Indonesia, Malaysia and Brunei. The genus was originally established to accommodate species formerly placed in *Puntius* s.l., a “catch-all” assemblage of Asian barbs, with *Desmopuntius hexazona* (Weber & de Beaufort, 1912) designated as the type species (Kottelat, 2013). Species presently recognised as *Desmopuntius* were originally assigned to either *Barbus* Daudin, 1805 or to *Puntius* Hamilton, 1822.

Recent molecular phylogenetic studies of the *Puntius* complex have provided strong support for the monophyly of the genus *Desmopuntius* (Katwate et al. 2020; Ren et al. 2020; Sudasinghe et al. 2023). These studies also demonstrated that the genus *Desmopuntius* is closely related to *Barbodes* Bleeker, 1859, *Oliotius* Kottelat, 2013, *Striuntius* Kottelat, 2013, *Puntigrus* Kottelat, 2013, *Hampala* Kuhl & van Hasselt, 1823, and *Waikhomia* Katwate, Kumkar, Raghavan & Dahanukar, 2020. Morphological characters that distinguish *Desmopuntius* from related genera include a distinctive colour pattern of 4–6 bars (at least in juvenile stage): the first across the eye, the second behind the gill opening, the third extending from the dorsal-fin origin, the fourth from anal-fin origin, the fifth on the caudal peduncle, and the sixth at the caudal-fin base. Additional diagnostic features include a black spot frequently present at the posterior end of the dorsal-fin base, the presence of both rostral and maxillary barbels, smooth and thin lips; and a postlabial groove that is medially interrupted (Kottelat 2013).

*Desmopuntius* currently comprises eight species: *D. foerschi* (Kottelat, 1982), *D. gemellus* (Kottelat, 1996), *D. hexazona*, *D. johorensis* (Duncker, 1904), *D. pentazona* (Boulenger, 1984), *D. rhomboocellatus* (Koumans, 1940), and *D. trifasciatus* (Kottelat, 1996), with *D. endecanalis* (Roberts, 1989) tentatively included (Kottelat 2013; see Discussion). These species occur in streams across the major Indonesian islands of Sumatra and Kalimantan.

Haryono (2005) examined species of the so-called “striped *Puntius*” group and reported an undescribed species referred to as *Puntius* sp. from East Kalimantan. Following further collection and examination of additional specimens, this species is herein recognised as a new species of *Desmopuntius* and formally described.

## ﻿Material and methods

The morphometric and meristic counts generally follow Kottelat (2001) and Kottelat and Freyhof (2007), with minor modifications and additional traits. Pectoral and pelvic-fin rays were counted as branched and unbranched; additional morphometrics included postorbital length, dorsal-fin height, anal-fin height, pectoral-pelvic distance, and pelvic-anal distance. Terminology of stripe patterns and scale-row numbering follows Kottelat (1996) and Kottelat and Lim (2021). Morphometric measurements were taken to the nearest 0.1 mm using a digital caliper, and are reported as a range of percentage of standard length (**SL**) or head length (**HL**). When multiple meristic values were observed, the modal value is given in parentheses. An asterisk (*) indicates a characteristic of the holotype. Total vertebral counts were obtained from radiographs. Meristic abbreviations used in this study are as follows: **A**, anal fin; **C**, caudal fin; **CPS**, circum-peduncular scale rows; **D**, dorsal fin; **P1**, pectoral fin; **P2**, pelvic fin; LL, lateral line scales; **TRa**, transverse scales from dorsal-fin origin to above the lateral line; **TRb**, transverse scales from below the lateral line to about two scales anterior to the pelvic-fin origin; **PreD**, predorsal scales; **GR**, gill raker count; and **VC**, vertebral count. Type specimens of the new species, as well as comparative material, are deposited in the Museum Zoologicum Bogoriense (**MZB**), Cibinong, Indonesia.

A Linear Discriminant Analysis (**LDA**) was conducted using PAST v. 5.2.1 (Hammer et al. 2001) to visualize the separation among *Desmopuntius* species. The analysis utilized all morphometric traits (as a percentage of SL) except for total length. In addition, a biplot analysis was employed to identify the primary morphometric characters that differentiate these species.

Total genomic DNA was extracted using a Geneaid DNA isolation kit following the manufacturer’s protocols. A pair of primers, FISH-FI (5’-TCAACCAACCACAAAGACATTGGCAC-3’) and FISH-RI (5’-TAGACTTCTGGGTGGCCAAAGAATCA-3’) (Ward et al. 2005), was used to amplify a partial fragment of the mitochondrial cytochrome *c* oxidase subunit 1 (*COI*) gene. PCR was carried out on an Eppendorf Mastercycler Nexus Gradient in a 25 µl reaction containing 15.5 µl sterile water, 5 µl 5x MyTaq reaction buffer, 0.5 µl MyTaq DNA polymerase, 0.5 µl of each primer (10 µM), and 2 µl genomic DNA. The thermal cycling profile consisted of an initial denaturation at 94 °C for 5 min, followed by 35 cycles of denaturation (94 °C for 30 s), annealing (50 °C for 40 s), and extension (72 °C for 60 s), with a final extension at 72 °C for 10 min. PCR products were electrophoresed on 2% agarose gel and visualized under ultraviolet transillumination. DNA sequencing was carried out using the same PCR primers on an ABI 3730XL DNA Analyzer at PT. Genetika Science Indonesia, Banten, Indonesia.

Chromatograms were assembled into bidirectional consensus sequences and manually edited using Bioedit v. 7.2 (Hall 1999). All newly generated sequences were submitted to GenBank (PV600755–PV600760). Additional sequences were downloaded from GenBank to construct a phylogenetic tree (Table 5). No tissues or *COI* sequences were available for *D. endecanalis*. Four species, *Barbodes binotatus* (Valenciennes, 1842), *Oliotius oligolepis* (Bleeker, 1853), *Striuntius lineatus* (Duncker, 1904) and *Waikhomia hira* Katwate, Kumkar, Raghavan & Dahanukar, 2020, were selected as outgroups. Genetic distances among *Desmopuntius* species were calculated based on the Kimura-2-Parameter (K2P) model in MEGA 11 (Tamura et al. 2021). A maximum-likelihood (ML) tree was generated in RAxML 8 (Stamatakis 2014) with 1000 bootstrap replicates, based on the GTR+I+G model selected by jModelTest 2 (Darriba et al. 2012). The resulting phylogenetic tree was visualized and edited in FigTree v. 1.3.1 (Rambaut 2009).

## ﻿Results

### ﻿Systematics

***Desmopuntius* Kottelat, 2013**

#### ﻿ Desmopuntius mahakamensis

Taxon classificationAnimaliaCypriniformesCyprinidae

Harefa, Haryono & Gustiano sp. nov.

42EFEDDE-8A45-5E0F-A8C1-ADE636009C32

https://zoobank.org/35B47D4B-DB66-4650-8D8D-5A0C2FC5D1B4

Figs 2, 3, 4, 5, Tables 1, 2

Puntius sp. — Haryono 2005: 56, fig. 2c.

##### Type material.

***Holotype*.** • MZB 6132, 53.2 mm SL; Indonesia: Borneo: Kalimantan Timur Province: Kutai Kertanegara Regency, Muara Wis Village (0°19'35.8"S, 116°29'37.7"E); H. Haryono; 03 July 1992. ***Paratypes* (*N* = 31)**. All from Indonesia: Borneo: Kalimantan Timur Province: • MZB 28243, 1 specimen, 52.8 mm SL, same data as holotype • MZB 17589, 11 specimens, 33.7–62.5 mm SL; small stream Belayan, tributary of Mahakam River; R.K. Hadiaty et al.; 25 April 2009 • MZB 19047, 5 specimens, 28.4–53.0 mm SL; small stream Long Tahap, tributary of Mahakam River; R.K. Hadiaty et al., 13 July 2010. • MZB 19076, 1 specimen, 30.5 mm SL, same data as for MZB 19047 • MZB 17327, 1 specimen, 32.7 mm SL; small stream Belayan; R.K. Hadiaty et al.; 15 April 2009 • MZB 17414, 1 specimen, 32.6 mm SL, small stream Long Tahap, tributary of Mahakam River, R.K. Hadiaty et al.; 19 April 2009. • MZB 28447, 11 specimens, 53.0–75.4 mm SL, Semayang lakes, Semayang Village (0°10'47.9"S, 116°26'46.2"E), T. Harefa; 29 March 2025.

##### Diagnosis.

*Desmopuntius mahakamensis* can be distinguished from all congeners by the following combination of characters: anal-fin rays 5½; lateral line scales 26–27 (mode 26); predorsal scales 9–11 (mode 10); gill rakers 9–11 (mode 10); total vertebrae 29; axial streak present; 5–6 black lateral stripes in adults (>30 mm SL), with stripe +1 extending from behind the gill opening along scale row 1, reaching only to the midbody, between the origin and end of the dorsal-fin base.

##### Description.

Morphometric and meristic data are presented in Tables 1, 3. Body laterally compressed and relatively deep, with greatest depth at dorsal-fin origin. Dorsal profile continuous from head to body, with a slight hump at the nape, ascending to dorsal-fin origin, then descending to the anterior base of the caudal fin. Ventral profile continues from head to body, slightly convex through the posterior end of the anal-fin base, and then slightly concave along the caudal peduncle. Rostral and maxillary barbels present. Snout blunt. Eyes large, almost aligned with the dorsal profile of the head. Mouth subterminal; maxilla extending to, or slightly beyond, the vertical of the anterior margin of the eye. Postlabial groove deep and interrupted medially. Lateral line complete, posteriorly descending for about six scales.

**Table 1.** Morphometric measurements of *Desmopuntius gemellus*, *D. johorensis*, *D. trifasciatus*, and *D. mahakamensis* sp. nov.

	*D. gemellus*		*D. johorensis*		*D. trifasciatus*		*D. mahakamensis* sp. nov.		
	*N* = 18		*N* = 11		*N* = 10		*N* = 15		
	range	mean (SD)	range	mean (SD)	range	mean (SD)	holotype	range	mean (SD)
Total length	61.5–81.0		88.8–125.6		60.5–102.4		68.1	56.7–82.8	
Standard length (mm SL)	47.6–65.1		69.2–93.9		46.1–77.6		53.2	43.0–62.5	
% in SL									
Head length	27.8–31.0	29.5 (0.8)	27.9–30.8	29.3 (0.9)	27.4–30.2	29.0 (0.9)	29.8	27.2–30.0	28.9 (0.8)
Predorsal length	50.8–56.4	53.0 (1.3)	48.3–51.2	50.1 (0.9)	48.4–53.6	51.5 (1.5)	51.4	45.8–52.3	50.2 (1.8)
Prepelvic length	50.3–57.6	53.3 (1.8)	48.3–51.5	50.1 (0.9)	47.7–53.1	50.4 (1.4)	48.2	47.5–51.5	49.8 (1.1)
Preanal length	74.2–78.9	76.4 (1.4)	68.2–74.9	72.6 (1.9)	72.5–77.3	74.9 (1.8)	72.3	70.2–75.1	72.8 (1.3)
Head depth	19.3–23.6	21.3 (0.9)	20.5–22.5	21.3 (0.5)	17.9–19.9	19.2 (0.6)	20.7	19.5–21.9	20.5 (0.8)
Body depth	32.7–38.4	36.0 (1.6)	33.9–40.3	35.7 (1.7)	28.6–32.8	30.7 (1.4)	36.0	31.1–36.6	34.5 (1.5)
Body width	14.2–18.6	16.7 (1.1)	15.8–18.8	17.1 (1.0)	11.7–16.7	14.4 (1.7)	17.1	14.1–17.7	16.1 (1.2)
Depth at caudal peduncle	12.5–14.3	13.6 (0.5)	12.8–14.8	13.8 (0.7)	11.6–14.0	12.7 (0.7)	13.8	13.2–15.5	14.0 (0.6)
Length at caudal peduncle	13.7–18.4	15.9 (1.4)	14.6–19.5	17.4 (1.7)	14.0–18.3	16.5 (1.3)	19.5	16.2–19.5	18.3 (1.0)
Snout length	6.7–9.2	7.8 (0.6)	6.4–8.4	7.4 (0.6)	6.8–8.1	7.5 (0.4)	7.3	5.4–7.8	6.5 (0.6)
Eye diameter	7.6–9.7	8.5 (0.5)	7.4–9.6	8.4 (0.7)	6.6–8.5	7.8 (0.5)	9.2	8.1–9.9	9.2 (0.5)
Interorbital width	9.9–11.2	10.6 (0.4)	10.0–11.5	10.9 (0.4)	8.7–11.3	9.6 (0.7)	9.5	9.0–11.4	10.0 (0.7)
Postorbital length	10.2–13.1	12.3 (0.8)	12.1–13.5	12.8 (0.5)	10.6–12.7	11.5 (0.7)	10.9	10.2–12.3	11.5 (0.6)
Dorsal-fin height	22.9–27.7	25.2 (1.6)	21.0–30.1	25.6 (2.3)	19.8–25.2	22.8 (1.7	24.6	24.0–28.8	26.8 (1.3)
Anal-fin height	12.3–15.6	14.1 (1.0)	14.5–16.7	15.9 (0.9)	11.7–14.9	13.3 (1.0)	16.1	16.0–18.7	17.3 (0.8)
Pectoral-pelvic distance	25.4–29.7	27.3 (1.2)	22.5–25.4	23.8 (0.9)	20.5–26.9	23.6 (1.9)	25.6	21.9–26.2	24.3 (1.2)
Pelvic-anal distance	22.6–29.0	24.9 (1.4)	22.9–25.9	24.9 (1.0)	21.2–26.2	23.7 (1.6)	22.7	22.3–25.1	23.3 (0.8)
Length of dorsal-fin base	12.8–17.2	15.3 (1.3)	16.0–18.7	17.6 (0.8)	14.8–17.2	15.9 (0.8)	17.1	15.9–17.8	16.8 (0.5)
Length of anal-fin base	8.1–10.5	9.5 (0.7)	10.3–11.7	10.9 (0.4)	8.5–11.5	9.9 (1.0)	10.1	9.9–11.2	10.5 (0.4)
Length of pelvic fin	17.1–21.1	19.5 (1.0)	19.5–24.2	21.0	18.7–22.2	19.9 (1.1)	20.9	20.3–23.7	21.9 (0.9)
Length of pectoral fin	18.4–22.1	20.2 (1.0)	20.6–24.2	21.6 (1.1)	16.6–22.7	20.4 (1.9)	21.1	20.3–24.9	22.6 (1.4)
Length of upper caudal lobe	27.2–34.6	30.9 (1.6)	28.8–33.7	32.0 (1.7)	31.6–38.0	33.0 (1.9)	34.5	30.3–37.8	34.7 (2.4)
Length of middle caudal rays	10.0–15.8	13.0 (1.3)	12.7–16.7	14.7 (1.3)	11.7–14.2	13.2 (0.8)	14.7	13.7–17.9	15.5 (1.3)
Length of lower caudal lobe	27.5–33.7	30.9 (2.2)	28.8–36.0	32.1 (2.1)	31.4–33.9	32.5 (0.7)	33.4	30.2–37.8	34.3 (2.2)
% in HL									
Snout length	23.1–30.0	26.6 (1.6)	23.1–29.7	25.1 (1.9)	22.9–29.3	26.0 (1.9)	24.6	19.4–24.6	22.4 (1.3)
Eye diameter	26.4–32.4	28.8 (1.7)	26.0–33.4	28.9 (2.8)	25.8–29.7	27.2 (1.4)	33.2	28.1–35.8	32.3 (2.0)
Interorbital width	33.6–38.0	36.0 (1.3)	33.6–39.5	37.3 (1.8)	29.4–37.8	33.0 (2.6)	32.0	31.4–38.3	34.4 (2.3)
Postorbital length	37.4–44.9	41.5 (2.4)	41.9–46.2	43.6 (1.2)	36.5–44.2	39.8 (2.5)	37.9	36.0–42.3	39.8 (2.2)

**Table 2.** Morphometric measurements of *Desmopuntius endecanalis*, *D. hexazona*, *D. pentazona*, and *D. rhomboocellatus*.

	*D. endecanalis*	*D. hexazona*		*D. pentazona*		*D. rhomboocellatus*	
	*N* = 1	*N* = 16		*N* = 9		*N* = 5	
	range	range	mean (SD)	range	mean (SD)	range	mean (SD)
Total length	49.6	42.0–56.2		38.0–43.7		38.3–59.1	
Standard length (mm SL)	48.2	32.0–43.9		27.9–32.0		29.5–42.7	
% in SL							
Head length	27.2	28.8–34.6	31.3 (1.4)	28.0–32.2	30.8 (1.2)	29.3–32.7	31.5 (1.5)
Predorsal length	51.2	45.4–54.7	49.9 (3.0)	47.9–53.5	51.4 (1.8)	47.8–50.0	48.9 (0.8)
Prepelvic length	45.9	42.9–48.5	46.2 (1.9)	44.9–52.7	49.3 (2.4)	48.2–50.0	49.0 (0.9)
Preanal length	67.8	68.6–75.2	72.2 (2.1)	70.4–78.0	73.5 (2.3)	71.3–74.9	73.0 (1.4)
Head depth	16.4	21.4–26.6	23.6 (1.3)	18.3–24.9	21.5 (2.4)	18.7–23.5	20.8 (2.1)
Body depth	35.4	41.5–47.8	44.5 (1.7)	36.4–41.6	39.2 (1.9)	34.1–43.2	38.1 (3.9)
Body width	13.6	13.9–18.7	16.1 (1.4)	12.4–17.2	14.6 (1.5)	12.5–17.0	14.7 (1.7)
Depth at caudal peduncle	14.2	16.0–18.1	17.2 (0.6)	14.8–15.8	15.5 (0.3)	13.4–15.5	14.7 (0.9)
Length at caudal peduncle	16.3	13.8–17.8	15.5 (1.3)	16.4–20.1	18.3 (1.1)	15.4–19.2	16.9 (1.5)
Snout length	6.6	6.3–8.8	7.4 (0.8)	6.4–8.5	7.1 (0.6)	6.3–7.3	6.9 (0.4)
Eye diameter	9.8	7.9–10.2	8.9 (0.6)	8.7–10.1	9.6 (0.5)	8.7–10.0	9.4 (0.5)
Interorbital width	7.4	9.9–11.3	10.6 (0.5)	5.6–11.0	(8.6 (1.9)	9.8–11.1	10.7 (0.5)
Postorbital length	10.5	11.8–14.4	13.1 (0.7)	12.0–14.9	12.8 (1.0)	11.8–14.1	12.8 (1.0)
Dorsal-fin height	17.4	20.6–29.5	25.6 (2.3)	22.2–29.6	26.4 (2.6)	25.0–27.6	25.9 (1.2)
Anal-fin height	13.8	14.5–18.6	17.2 (1.1)	17.2–19.0	18.1 (0.6)	14.0–18.9	16.2 (1.9)
Pectoral-pelvic distance	20.3	18.6–24.7	22.1 (1.8)	20.3–25.7	23.0 (1.4)	21.6–24.2	22.7 (1.3)
Pelvic-anal distance	20.7	23.8–28.9	26.3 (1.3)	22.9–27.3	25.5 (1.6)	20.5–24.8	23.4 (1.7)
Length of dorsal-fin base	16.3	18.2–22.8	21.1 (1.3)	18.2–20.2	19.1 (0.6)	17.5–20.0	18.9 (1.1)
Length of anal-fin base	13.8	10.5–13.2	12.3 (0.7)	7.9–12.0	10.2 (1.2)	9.4–12.8	11.1 (1.2)
Length of pelvic fin	21.0	22.4–26.2	24.3 (1.3)	21.0–27.2	23.8 (1.9)	19.5–25.0	21.0 (2.3)
Length of pectoral fin	18.3	19.7–26.0	23.2 (2.1)	21.7–24.2	22.8 (0.9)	18.3–24.0	34.9 (2.9)
Length of upper caudal lobe	-	29.0–40.1	35.2 (3.0)	34.0–38.3	36.3 (1.6)	31.4–39.3	34.9 (2.9)
Length of middle caudal rays	-	14.4–20.5	18.2 (1.8)	13.5–18.7	17.1 (1.5)	13.8–18.0	15.4 (1.8)
Length of lower caudal lobe	-	28.4–39.4	34.8 (3.2)	30.8–38.6	36.3 (2.5)	32.5–39.3	36.0 (2.4)
% in HL							
Snout length	16.3	20.5–26.6	23.5 (2.1)	21.3–27.4	23.2 (1.8)	19.5–24.5	21.9 (1.8)
Eye diameter	36.1	25.5–32.7	28.5 (1.7)	29.0–32.9	31.2 (1.2)	28.3–31.8	29.8 (1.4)
Interorbital width	27.2	31.2–37.6	34.0 (1.9)	29.4–35.5	32.1 (1.9)	30.1–36.8	34.0 (2.6)
Postorbital length	38.7	37.8–44.9	41.8 (2.2)	38.3–46.8	41.7 (2.8)	38.2–43.0	40.7 (2.2)

**Table 3.** Frequency distribution of meristic counts for *Desmopuntius* spp. examined in this study.

Species	D iv		A iii		P1 i					P2 i		TRa			TRb		CPS		PreD					LL							GR						VC			
	8½	M	5½	M	13	14	15	16	M	8	M	½4	½5	M	3½	M	12	M	8	9	10	11	M	22	23	24	25	26	27	M	7	8	9	10	11	M	29	30	31	M
***D. mahakamensis* sp. nov.**	32	8½	32	5½	-	22	10	-	14	32	8	32	-	½4	32	3½	32	12	-	1	29	2	10.0	-	-	-	9	23	-	25.7	-	-	2	8	3	10.1	9	-	-	29
*D. endecanalis*	1	8½	1	8½	-	1	-	-	14	1	8	1	-	½4	1	3½	1	12	-	1	-	-	9.0	-	-	1	-	-	-	24	-	-	1	-	-	9	-	-	1	31
*D. gemellus*	39	8½	39	5½	-	26	13	-	14	39	8	39	-	½4	39	3½	39	12	-	6	33	-	9.8	-	-	-	6	31	3	26.6	-	6	5	-	-	8.5	7	-	-	29
*D. hexazona*	16	8½	16	5½	1	11	4	-	15	16	8	11	5	½4.3	16	3½	16	12	-	9	7	-	9.4	5	9	2	-	-	-	22.8	2	6	4	1	-	8.3	3	1	-	29.3
*D. johorensis*	15	8½	15	5½		2	6	7	15	15	8	15	-	½4	15	3½	15	12	-	5	7	3	9.9	-	-	-	-	6	9	26.6	-	2	6	2	1	9.2	3	1	-	29.3
*D. pentazona*	10	8½	10	5½	1	7	2	-	14	10	8	10	-	½4	10	3½	10	12	7	3	-	-	8.3	4	6	-	-	-	-	22.6	2	6	2	-	-	8	2	-	-	29
*D. rhomboocellatus*	8	8½	8	5½	5	3	-	-	13	8	8	8	-	½4	8	3½	8	12	6	2	-	-	8.3	1	4	3	-	-	-	23.3	-	2	5	-	-	8.7	2	-	-	29
*D. trifasciatus*	19	8½	19	5½	-	-	8	11	16	19	8	19	-	½4	19	3½	19	12	-	3	15	1	9.9	-	-	-	2	15	2	26	-	5	4	3	1	9	3	1	-	29.3

Dorsal fin with 4 unbranched and 8½ branched rays; origin at about midbody, distal margin straight or slightly concave; last unbranched ray posteriorly serrated with 12–18 serrations. Anal fin with 3 unbranched and 5½ branched rays. Pectoral-fin rays with 1 unbranched and 14*–15 (modally 14) branched rays; fin rounded, first and second branched rays longest, not reaching pelvic-fin origin. Pelvic-fin with 1 unbranched and 9 branched rays; origin vertically behind dorsal-fin origin; unbranched ray longest, reaching only to anus. Caudal fin forked, lobes rounded to pointed at tips.

Lateral-line scales 25+2, 26+2* (mode 26+2), transverse scales ½4/1/3½ from dorsal-fin origin to about two scales anterior to pelvic-fin ray; predorsal scales 9–11 (mode 10*); circumpeduncular scales 12. Gill rakers 9–11. Total vertebrae 29.

***Colouration*.** Fresh specimens (Adult; Fig. 4). Dorsal side of the head dark brown, mid-lateral and ventral sides of the head greyish white. Small brown spots present below the eyes. Body with greyish-brown background, bearing 5–6 black lateral stripes: stripe 0 extending from just behind the gill opening, between scale row 0 and -1, to the middle of the caudal-fin base posteriorly; stripe +1 extending from behind the upper gill opening, along scale row +1, continuing only to midbody, between the origin and end of the dorsal-fin base; stripe +2 extending from the 5^th^–7^th^ of scale of row +2 to the upper caudal-fin base; stripe +3 extending from the nape along scale row +3 to slightly behind the end of the dorsal-fin base; stripe -1 extending from behind the gill opening, between scale rows -1 and -2, to the lower caudal-fin base; stripe -2, extending from behind the pectoral fin along scale row -3 to the anal-fin base, gradually decreasing in intesity anteriorly. Unbranched dorsal-fin rays dark brown, fin membrane yellowish-orange, fading posteriorly. Anal, pectoral, and pelvic fin membranes translucent. Caudal fin yellowish, becoming translucent posteriorly.

Preserved specimens (Adult; Fig. 2). Head with dark yellowish background, slightly darker dorsally, and pale yellowish ventrally. Body ground colour dark yellowish, becoming darker dorsally and pale yellowish ventrally, extending to below the dorsal fin, and in some specimens reaching just behind the gill opening anteriorly. Body with 5–6 black lateral stripes, in positions similar to those of live specimens. Axial streak present, posteriorly overlapping lateral stripe +1, extending anteriorly to below dorsal fin. All fins pale.

Juveniles (Fig. 3). Head and body with dark yellowish background. In specimens < 30 mm SL, body with three interrupted lateral stripes and 4–5 vertical dark bars or spot on the dorsal side: 1^st^ bar accros the eye; 2^nd^ bar extending behind the gill opening; 3^rd^ bar below the first and second branched ray of the dorsal fin, sometimes continuing to the pelvic fin; 4^th^ bar or spot at the end the of dorsal-fin base; 5^th^ bar or spot at the caudal penducle. In specimens > 30 mm SL, vertical bars usually faded, and lateral stripes form a more continuous pattern, except for stripe +1, which extends only to midbody.

##### Distribution.

**Figure 1.**
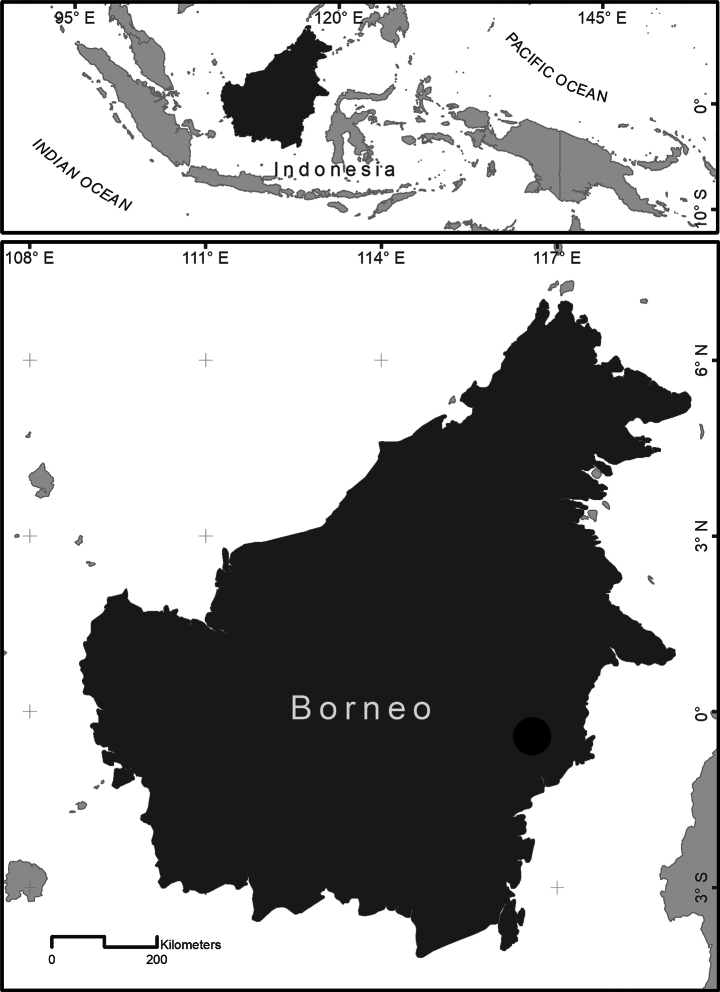
Map showing sampling site of *Desmopuntius mahakamensis* sp. nov. in Muara Wis, Kutai Kertanegara Regency, Kalimantan Timur Province, Borneo, Indonesia.

The new species is currently known only from the Mahakam River drainage, East Kalimantan, Indonesia. Most specimens have been collected from two lakes (Wis and Jempang) and from several small streams within the Mahakam River’s tributary system (Fig. 1).

**Figure 2.**
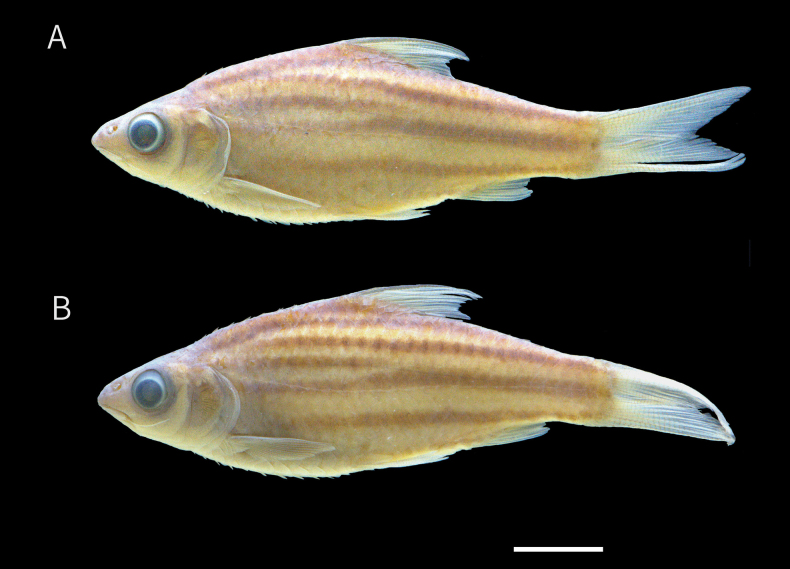
Preserved specimens of *Desmopuntius mahakamensis* sp. nov. A. Holotype, MZB 6132, 53.2 mm SL, Kalimantan Timur, Indonesia; B. Paratype, MZB 28243, 52.8 mm SL, same locality as holotype. Scale bar:10 mm.

**Figure 3.**
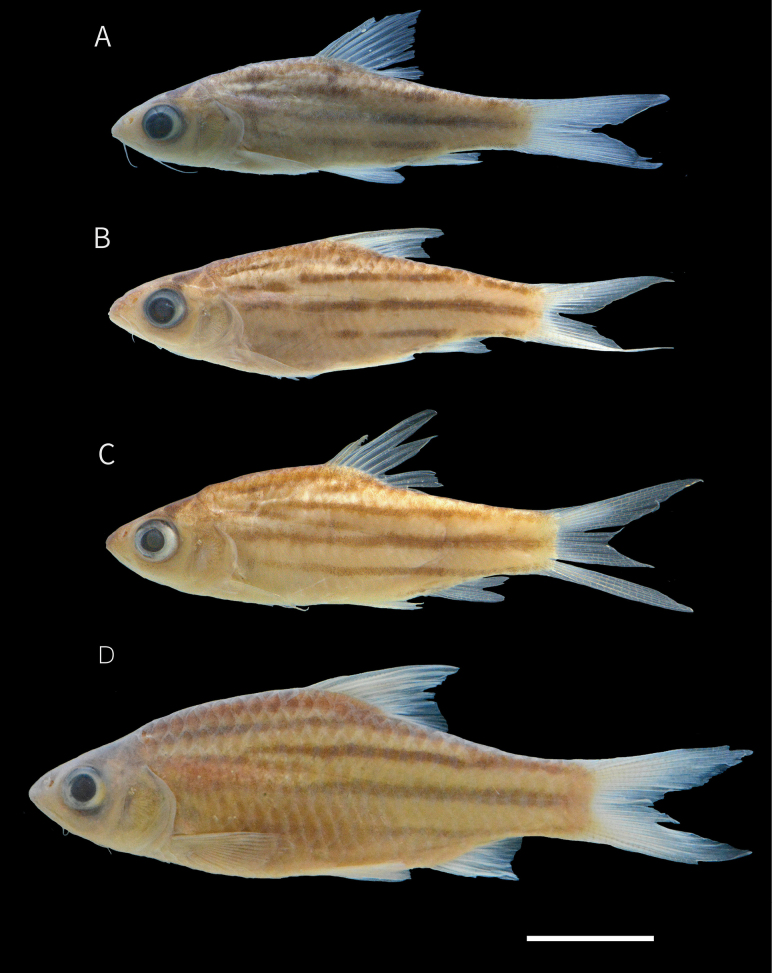
Preserved specimens of *Desmopuntius mahakamensis* sp. nov. A. Paratype, juvenile, MZB 19047, 28.4 mm SL; B. Paratype, MZB 19047, 30.5 mm SL; C. Paratype, MZB 17414, paratype, 32.6 mm SL; D. Paratype, MZB 19047 53.0 mm SL. Scale bar: 10 mm.

**Figure 4.**
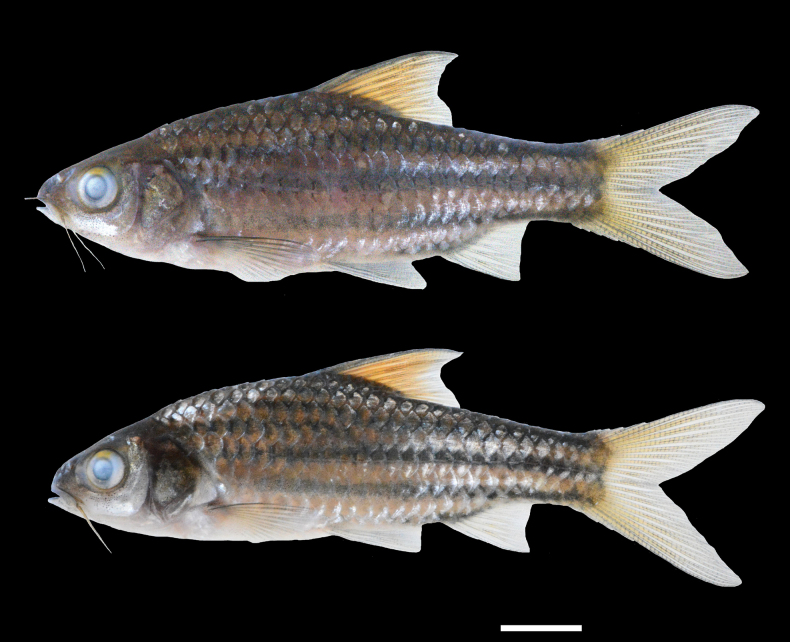
Fresh specimens of *Desmopuntius mahakamensis* sp. nov., paratypes, MZB 28247. A. 68.4 mm SL; B. 53.0 mm SL. Scale bar: 10 mm.

**Figure 5.**
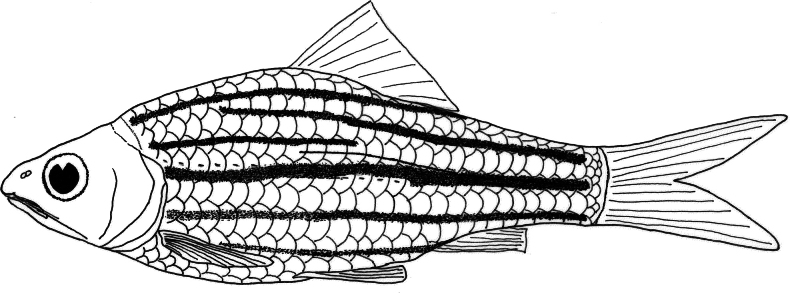
Schematic illustration of lateral pattern and interaxial streak of *D. mahakamensis* sp. nov., holotype, MZB 6232, 53.2 mm SL, Kalimantan Timur, Indonesia.

##### Etymology.

The specific name, *mahakamensis*, is derived from the Mahakam River, the locality where the species was collected.

##### Linear discriminant analysis (LDA).

The LDA of seven species of *Desmopuntius* showed that the first two functions explained 68.32% and 15.23% of the variance. Five morphometric traits were identified as primary characters distinguishing the species: head length, depth at the caudal peduncle, eye diameter, postorbital length, and length of the anal-fin base (Table 4). The LDA analysis showed that all currently represented species of *Desmopuntius* cluster into two morphological groups: one group includes *D. endecanalis*, *D. hexazona*, *D. pentazona*, and *D. rhomboocellatus*, while the other group includes *D. mahakamensis* sp. nov., *D. gemellus*, *D. johorensis*, and *D. trifasciatus* (Fig. 8).

**Table 4.** Factor loadings for variables in discriminant analysis. Values in bold indicate loadings > 0.80.

Measurements	Function 1	Function 2
Head length	0.413	**0.988**
Predorsal length	0.052	-0.016
Prepelvic length	-0.351	-0.076
Preanal length	-0.057	-0.106
Head depth	0.061	0.200
Body depth	0.134	0.390
Body width	-0.144	-0.333
Depth at caudal peduncle	**1.273**	-0.457
Length at caudal peduncle	-0.084	-0.328
Snout length	0.768	-0.060
Eye diameter	**1.904**	-0.003
Interorbital width	-0.340	-0.237
Postorbital length	-**2.452**	-0.251
Dorsal-fin height	-0.305	0.221
Anal-fin height	0.512	-0.361
Pectoral-pelvic distance	-0.091	0.174
Pelvc-anal distance	0.059	0.146
Length of dorsal-fin base	0.164	0.082
Length of anal-fin base	**0.976**	-0.276
Length of pelvic fin	0.298	-0.176
Length of pectoral fin	0.000	-0.149
Length of upper caudal lobe	-0.196	-0.358
Length of middle caudal rays	0.095	-0.003
Length of lower caudal lobe	0.083	0.062
Eigenvalues	36.51	8.14
% covariance	68.32	15.23

##### Genetic divergence and phylogenetic placement.

A total of 37 *COI* sequences were used for phylogenetic analysis (Fig. 9), including four newly generated sequences for *D. mahakamensis* and two for *D. gemellus*, each with a total length 651 bp. Among the *Desmopuntius* species studied, interspecific genetic divergence ranged from 0.2–15.2%, while intraspecific divergence ranged from 0.0–0.3%, based on the K2P model (Table 6). The mean genetic distance between *D. mahakamensis* and other congeners ranged from 7.2–13.0%, with *D. gemellus* being the most closely related species.

**Table 5.** Voucher code, GenBank accession numbers, sampling localities, and type localities of *Desmopuntius* species and outgroup taxa used in molecular analyses.

Species	Voucher	GenBank *COI*	DNA sample locality	Type locality	Source
*Barbodes binotatus*	BIF1655	MG699686	Indonesia: Java: Padeglang	Indonesia: Java: Bogor	
*Oliotius oligolepis*	RC0311	MN342655	Aquarium	Indonesia: Sumatra: Lake Maninjau	Collins et al. 2012
*Striuntius lineatus*	-	JF915641	Aquarium	Malaysia:Johor:Muar River	Collins et al. 2012
*Waikhomia hira*	BNHS FWF 603	MT103427	India: Chandewadi, Kamra	India: Chandewadi, Kamra	Katwate et al. 2020
*Desmopuntius foerschi*	RC0666	MN342492	Aquarium	Indonesia: Kalimantan: Sampit	Collins et al. 2012
*D. foerschi*	RC0100	MN342493	Aquarium	Indonesia: Kalimantan: Sampit	Collins et al. 2012
*D. foerschi*	RC0099	MN342494	Aquarium	Indonesia: Kalimantan: Sampit	Collins et al. 2012
*D. foerschi*	RC0098	MN342495	Aquarium	Indonesia: Kalimantan: Sampit	Collins et al. 2012
*D. foerschi*	RC0665	MN342496	Aquarium	Indonesia: Kalimantan: Sampit	Collins et al. 2012
*D. mahakamensis* sp. nov.	THR0521	PV600755	Indonesia: Kalimantan: Kutai Kertanegara	Indonesia: Kalimantan: Kutai Kertanegara	This study
*D. mahakamensis* sp. nov	THR0522	PV600756	Indonesia: Kalimantan: Kutai Kertanegara	Indonesia: Kalimantan: Kutai Kertanegara	This study
*D. mahakamensis* sp. nov	THR0523	PV600757	Indonesia: Kalimantan: Kutai Kertanegara	Indonesia: Kalimantan: Kutai Kertanegara	This study
*D. mahakamensis* sp. nov	THR0524	PV600758	Indonesia: Kalimantan: Kutai Kertanegara	Indonesia: Kalimantan: Kutai Kertanegara	This study
*D. gemellus*	-	MT483465	Indonesia: Sumatra: Way Kampas	Indonesia: Sumatra: Jambi	Ren et al. 2020
*D. gemellus*	-	MT483466	Indonesia:Air Lematang	Indonesia: Sumatra: Jambi	Ren et al. 2020
*D. gemellus*	THR0525	PV600759	Indonesia: Sumatra: Riau	Indonesia: Sumatra: Jambi	This study
*D. gemellus*	THR0526	PV600760	Indonesia: Sumatra: Riau	Indonesia: Sumatra: Jambi	This study
*D. hexazona*	RC0047	MN342497	Aquarium	Indonesia: Sumatra: Tuluk and Gunung Sahilan	Collins et al. 2012
*D. hexazona*	RC0046	MN342498	Aquarium	Indonesia: Sumatra: Tuluk and Gunung Sahilan	Collins et al. 2012
*D. hexazona*	RC0048	MN342499	Aquarium	Indonesia: Sumatra: Tuluk and Gunung Sahilan	Collins et al. 2012
*D. johorensis*	-	MT483467	Malaysia	Malaysia: Johor: Muar River	Ren et al. 2020
*D. johorensis*	RC0380	MN342500	Aquarium	Malaysia: Johor: Muar River	Collins et al. 2012
*D. johorensis*	RC0381	MN342501	Aquarium	Malaysia: Johor: Muar River	Collins et al. 2012
*D. johorensis*	RC0379	MN342502	Aquarium	Malaysia: Johor: Muar River	Collins et al. 2012
*D. johorensis*	RC0382	MN342503	Aquarium	Malaysia: Johor: Muar River	Collins et al. 2012
*D. johorensis*	RC0383	MN342504	Aquarium	Malaysia: Johor: Muar River	Collins et al. 2012
*D. johorensis*	RC0641	MN342505	Aquarium	Malaysia: Johor: Muar River	Collins et al. 2012
*D. pentazona*	RC0013	MN342506	Aquarium	Malaysia: Sarawak: Baram River	Collins et al. 2012
*D. pentazona*	RC0305	MN342507	Aquarium	Malaysia: Sarawak: Baram River	Collins et al. 2012
*D. pentazona*	RC0304	MN342508	Aquarium	Malaysia: Sarawak: Baram River	Collins et al. 2012
*D. pentazona*	RC0306	MN342509	Aquarium	Malaysia: Sarawak: Baram River	Collins et al. 2012
*D. ocellatus*	-	MT483468	Aquarium	Indonesia: Kalimantan: Banjarmasin	Ren et al. 2020
*D. rhomboocellatus*	RC0023	MN342512	Aquarium	Indonesia: Kalimantan: Banjarmasin	Collins et al. 2012
*D. rhomboocellatus*	RC0154	MN342512	Aquarium	Indonesia: Kalimantan: Banjarmasin	Collins et al. 2012
*D. rhomboocellatus*	RC0155	MN342512	Aquarium	Indonesia: Kalimantan: Banjarmasin	Collins et al. 2012
*D. trifasciatus*	-	MT483469	Aquarium	Indonesia: Kalimantan: Danau Sentarum	Ren et al. 2020
*D. trifasciatus*	-	MT483470	Aquarium	Indonesia: Kalimantan: Danau Sentarum	Ren et al. 2020

**Table 6.** Pairwise *COI* genetic distances for eight *Desmopuntius* species.

	*D. mahakamensis*	*D. foerschi*	*D. gemellus*	*D. hexazona*	*D. johorensis*	*D. pentazona*	*D. rhomboocellatus*	*D. trifasciatus*
*D. mahakamensis*	0.000							
*D. foerschi*	0.130	0.001						
*D. gemellus*	0.072	0.123	0.002					
*D. hexazona*	0.122	0.138	0.128	0.000				
*D. johorensis*	0.095	0.150	0.095	0.143	0.001			
*D. pentazona*	0.123	0.101	0.126	0.152	0.143	0.003		
*D. rhomboocellatus*	0.125	0.111	0.112	0.096	0.130	0.142	0.000	
*D. trifasciatus*	0.095	0.150	0.096	0.142	0.002	0.144	0.130	0.003

The topology of the ML tree strongly supported the monophyly of the genus *Desmopuntius*. Within the genus, two major clades were recognised with moderately high bootstrap support (BP: 78). The first clade, the “*D. hexazona* group,” comprised four species: *D. hexazona*, *D. rhomboocellatus*, *D. pentazona*, and *D. foerschi*. The second clade, the “*D. johorensis* group,” included *D. mahakamensis*, *D. gemellus*, *D. johorensis*, and *D. trifasciatus*. Except for the node between *D. hexazona* and *D. rhomboocellatus*, most species-level nodes within *Desmopuntius* had poorly supported bootstrap values (BPs: < 70). In addition, *D. trifasciatus* was nested within *D. johorensis* (BP: 100), and the new species *D. mahakamensis* was found to be closely related to *D. gemellus* (BP: 54).

## ﻿Discussion

The genus *Desmopuntius* currently comprises nine species, including the newly described species presented here. Kottelat (2013) tentatively included *D. endecanalis* in *Desmopuntius*, an assignment followed in this study. *Desmopuntius endecanalis* differs from other species in the genus in having a greater number of branched anal-fin rays (iii,7–8½ vs. iii,5½) (Roberts 1989). The new species is further distinguished from *D. endecanalis* by having fewer vertebrae (29 vs. 31), more lateral line scales (25–26 vs. 24), a shorter anal-fin base (9.9–11.2 vs. 13.8% of SL), and a different colour pattern (adults with 5–6 black lateral stripes vs. 5–6 black vertical stripes).

**Figure 6.**
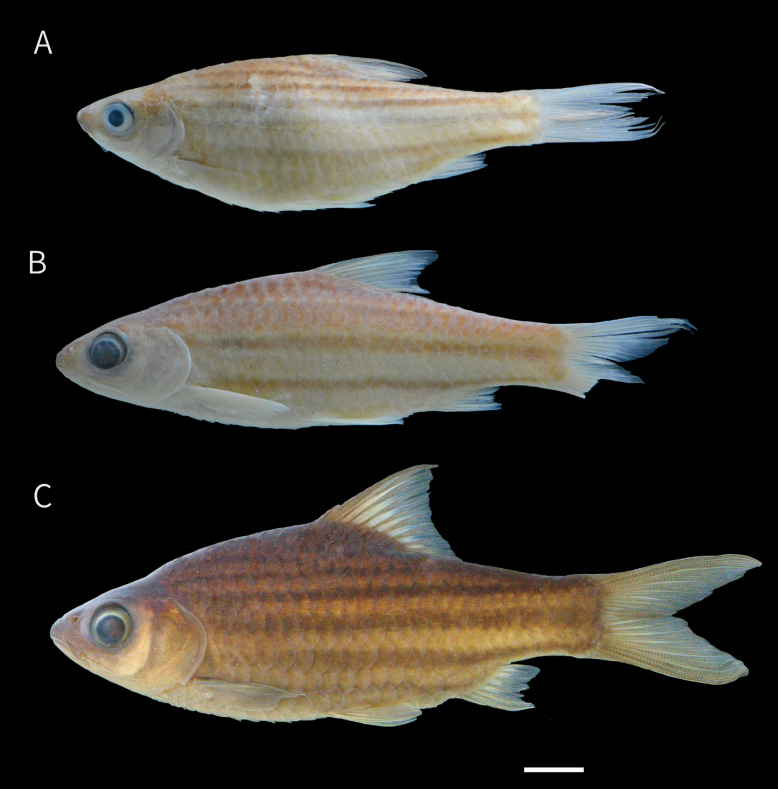
Preserved specimens of three *Desmopuntius* species possessing a lateral stripe pattern. A. *D. gemellus*, holotype, MZB 5939, 65.0 mm SL; B. *D. trifasciatus*, holotype, MZB 5940, 74.0 mm SL; C. *D. johorensis*, non-type, MZB 4184, 89.1 mm SL. Scale bar: 10 mm.

**Figure 7.**
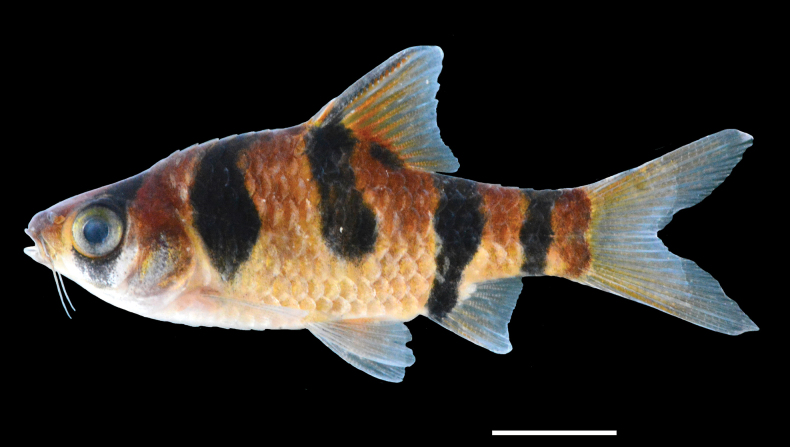
*Desmopuntius rhomboocellatus*, MZB 28448, 43.9 mm SL, Sebangau, Palangkaraya City, Kalimantan Tengah, Indonesia. Scale bar: 10 mm.

The new species *D. mahakamensis* is assigned to the *D. johorensis* group, which comprises *D. johorensis*, *D. gemellus* and *D. trifasciatus* (see Fig. 6). This group is characterised by a colour pattern of 3–6 black lateral stripes on the body in adults, whereas members of the *D. hexazona* group (*D. endecanalis*, *D. foerschi*, *D. hexazona*, *D. pentazona*, and *D. rhomboocellatus*) display 5–6 black vertical bars (see Fig. 7). In addition to the colour pattern, the new species can be readily distinguished from members of the *D. hexazona* group, except *D. endecanalis*, by a combination of meristic and morphological characters: from *D. foerschi* by having more predorsal scales (modally 10 vs. 9), greater body depth (31.1–36.6 vs. 29.8–32.2% of SL), and greater caudal-peduncle depth (13.2–15.5 vs. 12.0–13.1% of SL) (Roberts 1989); from *D. hexazona* by having more lateral line scales (25–26 vs. 22–24), lower head depth (19.5–21.9 vs. 21.4–26.6% of SL), lower body depth (31.1–36.6 vs. 41.5–47.8% of SL), and lower caudal-peduncle depth (13.2–15.5 vs. 16.0–18.1% of SL); from *D. pentazona* by having more lateral line scales (25–26 vs. 22–23), more predorsal scales (modally 10 vs. 8), more gill rakers (modally 10 vs. 8), and lower body depth (31.1–36.6 vs. 36.4–41.6% of SL); from *D. rhomboocellatus* by having more lateral-line scales (25–26 vs. 22–24), more predorsal scales (modally 10 vs. 8), and shorter dorsal fin-base (15.9–17.8 vs. 17.5–20.0% of SL).

*Desmopuntius mahakamensis* can be readily differentiated from *D. trifasciatus* and *D. johorensis* by the presence of an axial streak (vs. absent) (Fig. 5). It further differs from *D. trifasciatus* in having more lateral stripes (5–6 vs. 3–4), and from *D. johorensis* by a shorter snout length (19.4–24.6 vs. 23.1–29.7% of HL). In addition, it further differs from *D. trifasciatus* in having a greater head depth (19.5–21.9 vs. 17.9–19.9% of SL), a deeper body (31.1–36.6 vs. 28.6–32.8% of SL), and a larger eye diameter (28.1–35.8 vs. 25.8–29.7% of HL).

*Desmopuntius mahakamensis* is most similar to *D. gemellus*, a species recorded from Sumatra (Jambi, Riau, and Bangka) and possibly central Kalimantan (Kottelat 1996), in possessing an axial streak and 5–6 lateral stripes. However, the new species can be distinguished by a stripe +1 that extends from behind the upper gill opening to the midbody, between the beginning and end of the dorsal-fin base (vs. stripe +1 extending posteriorly to the upper caudal-fin base in *D. gemellus*). In addition, *D. mahakamensis* differs from *D. gemellus* in having a shorter snout (19.4–24.6 vs. 23.1–30.0% HL), a shorter predosal length (45.8–52.3 vs. 50.8–56.4% SL), a shorter prepelvic length (47.5–51.5 vs. 50.3–57.6% SL), and a shorter preanal length (70.2–75.1 vs. 74.2–78.9% SL).

A threshold genetic distance of > 2% is generally adopted for taxonomic identification (Hebert et al. 2004; Ward et al. 2005). The present study revealed a clear separation and genetic differentiation between *D. mahakamensis* and other *Desmopuntius* species (7.2–13.0%), although *D. trifasciatus* was found nested within *D. johorensis*. These values are comparable to those reported for other freshwater fishes in the Asian region, based on *COI* sequences, for example, among *Parachela* species (interspecific divergence 3.9–14.1%) (Page et al. 2024) and among *Rasbora* species in Sri Lanka (2.0–12.1%) (Sudasinghe et al. 2020).

**Figure 8.**
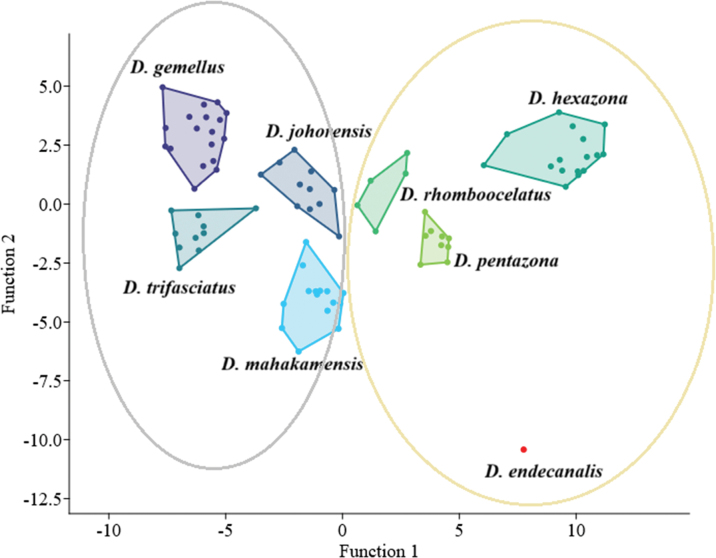
Linear Discriminant Analysis (LDA) plot of morphometric data for *Desmopuntius* spp.

The current molecular data showed no distinction between *D. johorensis* and *D. trifasciatus*, suggesting a potential misidentification in previous studies. However, the two species can be reliably differentiated by morphological differences, particularly the number of lateral black stripes: *D. johorensis* with 5–6 stripes, versus *D. trifasciatus* with 3 stripes. Most species-level nodes within the *Desmopuntius* phylogeny showed low bootstrap support, potentially due to insufficient sequence length. In contrast, the current analysis of morphological data clearly distinguished all *Desmopuntius* species, although *D. foerschi* was not included in the analysis (Fig. 8). In light of these findings, we recommend a broader genetic dataset, incorporating additional taxa, wider geographic sampling, and multiple molecular markers, including both mitochondrial and nuclear sequences.

**Figure 9.**
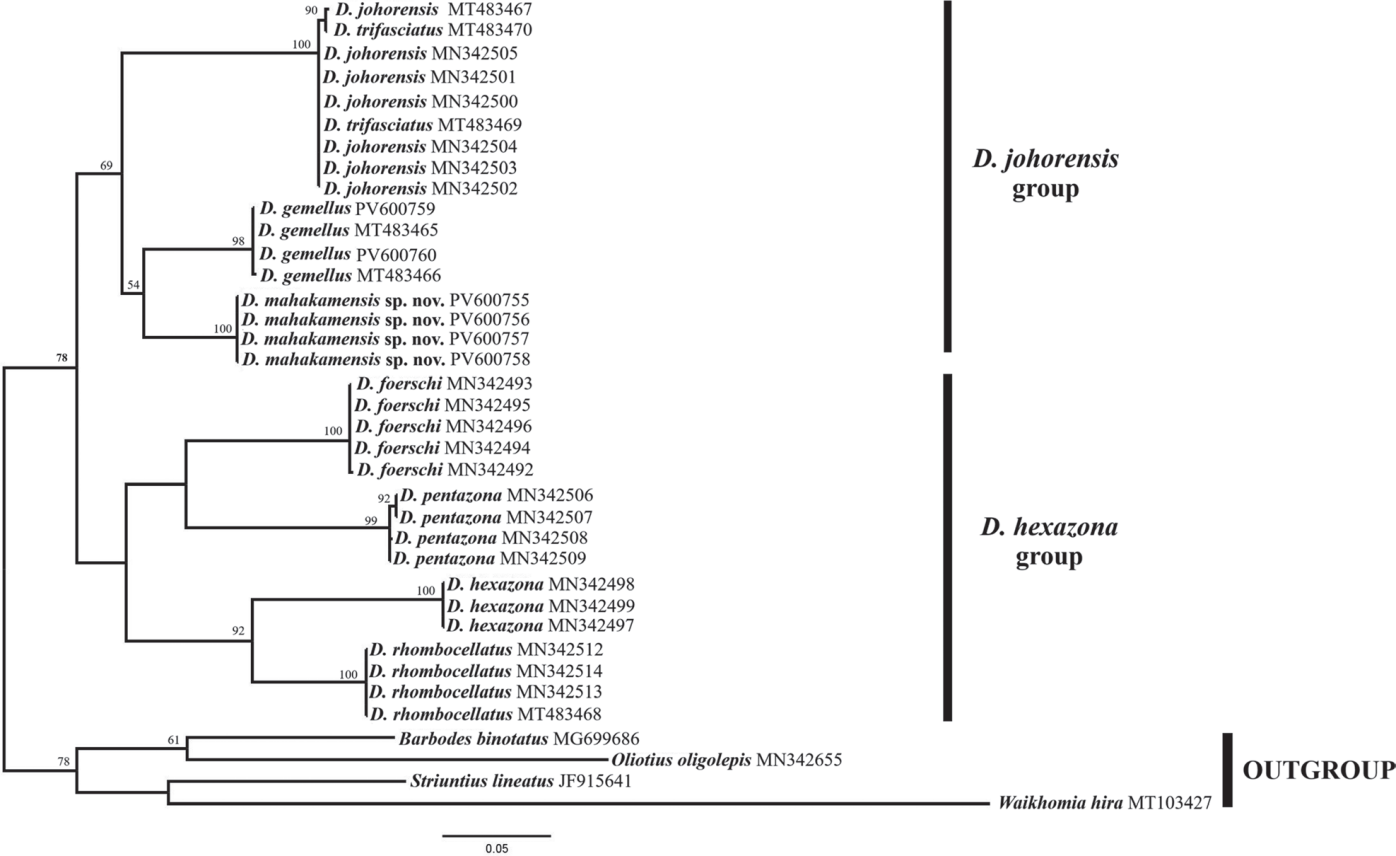
Maximum-likelihood (ML) phylogeny of *Desmopuntius*. Bootstrap values (> 50) are shown at the nodes.

### ﻿Key to species of *Desmopuntius*

**Table:** 

1	Body with 3–6 lateral stripes in adults	**2**
–	Body with 4–5 vertical stripes in adults	**5**
2	Interaxial streak present	**3**
–	Interaxial streak absent	**4**
3	Body with 5–6 lateral stripes; stripe +1 extending from behind gill opening on scale row +1, continuing between rows +1 and +2, reaching upper caudal-fin base posteriorly	***D. gemellus***
–	Body with 5–6 lateral stripes; stripe +1 extending from behind gill opening on scale row 1, reaching only midbody, between origin and end of dorsal-fin base	***D. mahakamensis* sp. nov.**
4	Body with 3–4 lateral stripes; stripes +1 and -1, extending along scale rows +3 and -3, reaching both upper and lower caudal-fin base posteriorly	***D. trifasciatus***
–	Body with 5–6 lateral stripes; stripes +1 and -1, extending along scale rows +2 and -2, reaching both upper and lower caudal-fin base posteriorly	***D. johorensis***
5	Anal-fin rays iii,7–8½	***D. endecanalis***
–	Anal-fin rays iii, 5½	**6**
6	Body with black spot between bars along the midline	***D. foerschi***
–	Body without black spot between bars	**7**
7	First and second vertical stripes on the body wide, terminating in a rounded or circular pattern	***D. rhomboocellatus***
–	First and second stripes on the body narrow, do not terminate in a rounded or circular pattern	**8**
8	Body with 5 vertical stripes; final stripe at caudal-fin base notably prominent	***D. hexazona***
–	Body with 4 vertical stripes; no stripe at caudal-fin base	***D. pentazona***

## Supplementary Material

XML Treatment for Desmopuntius mahakamensis

## ﻿Additional information

### Use of AI

No use of AI was reported.

### Author ORCIDs

Tonisman Harefa https://orcid.org/0000-0003-2308-8669

Haryono Haryono https://orcid.org/0000-0002-5143-6790

Rudhy Gustiano https://orcid.org/0000-0001-7205-0101

Tedjo Sukmono https://orcid.org/0000-0002-2946-9780

Gema Wahyudewantoro https://orcid.org/0000-0002-4819-4487

### Data availability

All of the data that support the findings of this study are available in the main text.

## ﻿Appendix 1: Comparative materials

***Desmopuntius endecanalis***: Indonesia: Kalimantan • MZB 4003, holotype, 48.2 mm SL; small forest stream where it flows into the sungai Mandai tributary 2–3 km upstream from its confluence with Kapuas mainstream, 17 km west-southwest of Putussibau (Kapuas 1976-37), Kalimantan Barat Province; T.R. Roberts, 1976.

***Desmopuntius gemellus***: Indonesia: Sumatra: • MZB 5939, holotype, 65.0 mm SL; market in Jambi; P.G. Bianco & M. Kottelat; 09 December 1984 • MZB 1390, 38 specimens 43.7–65.1 mm SL; Way Perdamaian, Lampung; Wargasasmita, S., 12 Februari 1972 • MZB 4243, 1 specimen, 59.9 mm SL; Lubuk Lampan, Lempeing River; Hartoto, D.I.; 4 October 1981.

***Desmopuntius hexazona***: Indonesia: Sumatra: Riau: Bengkalis: • MZB 2600, 4 specimens, 33.1–40.4 mm SL; Pinang Sebatang, Siak Indrapura; Haryono; 14 January 1996 • MZB 2630, 9 examined of 16 specimens; same data as for preceding • MZB 6225, 15 specimens, 22.9–32.9 mm SL; along road leading in to Tanjung, Bintan, 1°07'18.6"N, 104°25'58.7"E; S.H Tan & H.H. Tan; 27 June 1995 • MZB 6746, 1 specimen, 36.3 mm SL; Sungai Bukit Kapur Plintung Village, Bukit Kapur District; A.H. Tjakrawidjaya; 14 July 1996 • MZB 6766, 3 specimens, 38.5–42.0 mm SL; Plintung River, Bukit Kapur District; Tjakrawidjaya, A.H.; 15 July 1996.

***Desmopuntius johorensis***: Indonesia: Sumatra: • MZB 2534, 1 examined of 6 specimens; 31.6–59.3; Pinang Sebatang, Bengkalis, Riau; Haryono; 7 October 1999 • MZB 2593, 1 specimen, 90.2 mm SL; Lubuk Lampam, Lempuing River, Sumatra Selatan; unknown collector; 04 October 1981 • MZB 4184, 8 specimens 69.2–93.9 mm SL, Air Helam River, outside Simpang Gajah, Berbak; unknown collector; 27 April 1980 • MZB 6745, 2 examined of 3 specimens 69.4–75.8 mm SL, Plintung River, Plintung village, Bukit Kapur, Bengkalis, Riau; Agus H.T.; 15 July 1996 • MZB 23833, 3 specimens, 61.7–78.5 mm SL; Serkap River, Semenanjung Kampar, Teluk Meranti, Riau; Musadat et al.; 27 January 2017.

***Desmopuntius pentazona***: • MZB 12190, 9 specimens, 24.9–30.6 mm SL; Air Merah River, Kuala Raya village, Singkep Barat, Riau Archipelago, Indonesia; S. Sauri & A. Hamid; 22 May 2003 • MZB 17431, 1 specimen, 27.9 mm SL; Belayan River, Kembang Janggut District; Kalimantan Timur Province, Indonesia; R.K. Hadiaty et al.; 19 April 2009.

***Desmopuntius rhomboocellatus***: Indonesia: Kalimantan: • MZB 3302, 1 specimens 31.5 mm SL; small stream of Sungai Penyu, Pontianak, Kalimantan Barat; T.R. Roberts & Soetikno; 13 July 1976 • MZB 5331, 2 specimens 39.1–42.7 mm SL; Ampalit River, Katingan Ilir Kotim District, Kalimantan Tengah Province; Saim A.; 20 March 1984, MZB 28448, 4 specimens, Sebangau, Palangkaraya City, Kalimantan Tengah Province; T. Harefa; 27 March 2025.

***Desmopuntius trifasciatus***: Indonesia: Kalimantan: • MZB 5940, holotype, 74.0 mm SL; Nanga Semunak (Dry Season collection), Danau Sentarum Wildlife Reserve, Kapuas Basin, Kalimantan Barat; M. Kottelat et al.; 8 September 1993 • MZB 2595, 10 specimens 41.9–48.9 mm SL; Putat River, Danau Bekat, Tayan, Sanggau; R.K. Hadiaty & A. Mun’im; 22 November 1992 • MZB 3292, 1 specimen, 36.4 mm SL; Tekan River, ± 5–6 km from Sanggau; T.R. Roberts & Soetikno, 16 August 1976 • MZB 3294, 1 specimen, 77.6 mm SL; Webian River, Kalimantan Barat; T.R. Robert & Soetikno, 19 July 1976 • MZB 5465, 5 specimens, 63.2–74.0 mm SL; Kapuas River, Sintang, Kalimantan Barat Province; Hardjono, D., 24 September 1981. • MZB 18575, 1 specimen, 80.6 mm SL; Danau Siawan, Pontu Village, Kalimantan Barat Province; Agus, H.T. et al.; 20 December 2009.
